# Biological and Clinical Rationale for Androgen Priming in Ovarian Stimulation

**DOI:** 10.3389/fendo.2020.00627

**Published:** 2020-09-04

**Authors:** Kristine Løssl, Nina la Cour Freiesleben, Marie Louise Wissing, Kathrine Birch Petersen, Marianne Dreyer Holt, Linn Salto Mamsen, Richard A. Anderson, Claus Yding Andersen

**Affiliations:** ^1^The Fertility Clinic, Copenhagen University Hospital, Rigshospitalet, Copenhagen, Denmark; ^2^The Fertility Clinic, Copenhagen University Hospital Hvidovre, Hvidovre, Denmark; ^3^Alleris-Hamlet Fertility, Søborg, Denmark; ^4^VivaNeo Denmark, Copenhagen, Denmark; ^5^Zealand Fertility Clinic, Zealand University Hospital, Køge, Denmark; ^6^Laboratory of Reproductive Biology, The Juliane Marie Centre for Women, Children and Reproduction, University Hospital of Copenhagen, Copenhagen, Denmark; ^7^MRC Centre for Reproductive Health, University of Edinburgh, Edinburgh, United Kingdom; ^8^Faculty of Health and Medical Sciences, University of Copenhagen, Copenhagen, Denmark

**Keywords:** androgen priming, testosterone, follicular responsiveness, follicular recruitment, local androgen production, LH activity, IVF

## Abstract

Androgen receptors are expressed by all stages of growing follicles, and follicular fluid androgen levels are positively correlated to granulosa cell androgen receptor and follicle-stimulating hormone (FSH) receptor expression. Thus, androgens may promote follicular growth, accumulation and/or responsiveness to gonadotropins. This is explored therapeutically in the concept of androgen priming, to improve the ovarian response to stimulation in assisted reproduction. Androgen effects may be achieved in two different ways, either directly by providing exogenous androgen or by providing luteinizing hormone (LH) activity [i.e., LH or human chorionic gonadotropin (hCG)] to stimulate local ovarian production of androgen. The androgen concentrations in follicular fluid by far exceed the levels in female circulation and it has recently been shown that there was no correlation between serum testosterone levels and follicular fluid androgen levels. There is some evidence that administration of exogenous dehydroepiandrosterone or testosterone increases live birth rates, but an optimal protocol has not been established and such adjuvant treatment should be considered experimental. Furthermore, studies exploring long-term administration of LH activity, achieving LH levels comparable to those seen in women with polycystic ovary syndrome, are awaited. The aim of the present review is to discuss critically the most suitable approach for androgen priming from a biological and clinical standpoint, and to evaluate current approaches and results obtained in clinical trials.

## Capsule

Androgen priming has a biological basis for improving poor response during ovarian stimulation. While administration of exogenous androgens may have effects on early stages of follicle development, they are unlikely to affect the androgen concentrations in more mature follicles. Conversely, administration of hormones that increases the intra-follicular androgen concentration may have effects across a broader range of stage of follicle development.

## Introduction

Androgens play key roles in a range of reproductive functions necessary for conception, acting directly through the androgen receptor (AR), or as necessary precursors for estrogen synthesis. Androgens are considered to represent a double-edged sword in human follicular development, with positive effects on follicles at preantral and small antral stages and potential negative effects on pre-ovulatory follicles unless appropriate downregulation of granulosa cell AR takes place (1, 2). Women with polycystic ovary syndrome (PCOS) are characterized by a high antral follicle count and increased androgen levels, and androgens seem to play an important role in aberrant follicle development and anovulation in this group of women (3, 4). The pathophysiology of PCOS is however complex: although testosterone levels in small follicles from women with PCOS are increased and luteinized granulosa cells showed significantly increased transcripts of FSHR in PCOS, CYP19 expression is reduced, thus the follicular hyperandrogenism downregulates conversion to estrogen (5, 6); these alterations may impact on oocyte quality (7). Conversely, androgens may enhance follicular responsiveness to FSH by an upregulation of granulosa cell FSH receptor (FSHR) expression. In human small antral follicles (3–9 mm) a positive correlation between granulosa cell FSHR expression and both follicular fluid androgen levels and granulosa cell AR expression was demonstrated (8). Thus, at early stages, androgens may promote follicle growth, and this might be exploitable therapeutically.

In the human ovary, AR gene expression has been detected in preantral follicles from the primary stage onwards (9) and in antral follicles peaking at ~6 mm (10). AR protein has been observed in follicles from the primordial stage, gradually increasing during follicle development so that it is present in all multi-layered preantral follicles (2, 11). The duration of follicular growth from the multi-layered preantral stage to the stage where cyclic recruitment toward dominance and ovulation occurs (antral follicles of 2–5 mm) is estimated to last ~70 days (12). Therefore, this period may include developmental stages where follicles are sensitive to androgens, which may result in increased granulosa cell FSHR expression thus enhancing the number of follicles responding to exogenous FSH administration.

One of the most challenging groups of patients in infertility treatment is women with diminished ovarian reserve (DOR), who only develop a limited number of follicles after ovarian stimulation (OS) and have reduced chances of becoming pregnant (13). This group of patients is becoming more prevalent due to general societal changes in postponing childbearing. The possibility of developing improved stimulation protocols to increase the number of oocytes harvested following OS in such patients has received huge interest and investigation in recent years. Androgen priming in women with DOR has been investigated, but without consensus on how it should be performed or indeed whether it is an effective approach.

The aim of the present review is to discuss critically the most suitable approach for androgen priming based on a biological rationale, and to evaluate current approaches and results obtained in clinical trials.

## Biological Rationale of Androgen Priming

The widespread expression of AR in the ovary (2) indicates a likely range of actions and complicates interpretation of specific effects of androgens on follicular development. In AR knock out (ARKO) studies in rodents, global AR-null mice showed aberrant folliculogenesis with lower numbers of antral follicles and fewer oocytes after stimulation, they were sub-fertile and developed premature ovarian insufficiency (14, 15). Mice that specifically lacked AR expression in the granulosa cells had affected reproduction similarly to the global ARKO (AR-null mice) with fewer ovulated oocytes, resulting in 1.6 pups per litter compared to 6.6 in wildtype (14). Their ovaries contained a different distribution of follicle classes with fewer antral follicles but more preantral and atretic follicles as compared to wildtype mice. Further, *in vitro* growth of isolated preantral follicles from ARKO mice were slower than that of wildtype and number of litters per female was halved as they developed premature ovarian insufficiency. These studies in mice clearly point to the granulosa cells as targets of the main androgen effect on ovarian function and reproductive success and justify focusing on the granulosa cell as the key target for androgen action in the ovary.

Landmark studies in primates now two decades old showed that testosterone administration had a pronounced effect on follicles and granulosa cells after only 3- and 10-days administration (16–19). These studies demonstrated that granulosa cell AR expression was significantly increased after testosterone administration and that AR expression was positively associated with expression of the proliferation-specific antigen Ki-67 and negatively associated with granulosa cell apoptosis (17). Testosterone exposure increased the number of growing follicles (indicating an effect on the earliest stages of folliculogenesis) and granulosa cell responsiveness to FSH. Equally important, individual follicles demonstrated a significant positive association between FSHR and AR mRNA levels irrespective of testosterone administration. Furthermore, testosterone administration significantly increased granulosa cell FSHR expression. It was suggested that testosterone promotes follicular growth by amplifying the effect of FSH.

In those primate studies testosterone was administered in two doses (i.e., 4 or 0.4 mg/kg), which when extrapolated to a woman with a weight of 65 kg would correspond to doses of 260 and 26 mg/day, respectively, which are far beyond the threshold for inducing virilizing effects in women and therefore not clinically relevant. By comparison, 5–10 mg testosterone/day is required for physiological replacement in adult men.

Further strengthening the association between FSH sensitivity and androgens, evaluation of follicular fluid and the associated granulosa cells from normal human small antral follicles found highly significant associations between FSHR expression and both AR expression in granulosa cells and follicular fluid androgen levels (10). Thus, these data support a close link between FSHR expression and the action of androgens within small antral follicles in normal women, and support the hypothesis that androgens drive normal follicular development, and that this occurs across a broad range of stages of follicle development.

Taken together, there is therefore a clear biological basis for exploring the concept of androgen priming and the potential of recruiting an increased number of follicles in respond to OS. What is less clear is how this androgen priming is best achieved and how the development of clinical applicable methods should be addressed.

## Production of Androgens and Effects of Androgen in the Human Ovary Including Human Small Antral Follicles

It is well-established that ovarian androgens are produced by the theca cells under appropriate stimulation of LH, or hCG. Androgens are aromatized into estrogens within granulosa cells. Androgen production is critically dependent on the expression of the CYP17 enzyme that during a two-step reaction catalyzes conversion of pregnelonone to DHEA *via* 17-OH-pregnelonone. Androstenedione production by human theca cells *in vitro* was augmented 20-fold by the presence of inhibin A and insulin-like growth factor-1 (IGF1) in the culture medium (20). The highest concentration of inhibin tested was 100 ng/ml, which has now been shown to be low compared to that found in the follicular fluid of human small antral follicles (21, 22). Since inhibin A and inhibin B are only produced by granulosa cells and not the theca cells, after stimulation with FSH, this clearly demonstrates that follicular androgen production is under the control of both LH and FSH, involving an interaction between theca and granulosa cells in a development of the 2 cell, 2 gonadotrophin model of follicle function.

Recently, concentrations of Inhibin B in fluid from normal small antral follicles obtained in natural menstrual cycles were associated with follicular fluid concentrations of androgens and to gene expression levels in granulosa cells (23). Inhibin B concentrations were strongly positively associated with intrafollicular concentrations of testosterone and androstenedione and with mRNA levels of FSHR, LHR, and CYP19, while AR mRNA was not related (23). These data strengthen the notion that inhibins also play an important role in the regulation of androgen synthesis *in vivo*, which then in turn up-regulates FSHR. Downstream, this upregulates LHR and aromatase (i.e., CYP 19) expression in granulosa cells.

Recent data suggests that AR expression is under the control of LH, as *in vitro* studies on human granulosa cells found a significant down regulation of AR in the presence of LH (4). However, LHR only starts to become expressed in granulosa cells around the time of follicular selection at a diameter of 8–10 mm in normal women, and the regulation of AR expression in earlier stages of human folliculogenesis has not been described.

## Clinical Aspects of Androgen Priming

Increased androgen levels may be achieved in two different ways, either by the administration of exogenous androgen (i.e., DHEA or testosterone) or by providing hormones that stimulate enhanced local production of androgen within the ovaries (i.e., LH or hCG). Alternatively, estrogen production may be blocked by the administration of an aromatase inhibitor (AI) causing androgen accumulation. Administration of LH, hCG, or AI has likely advantages in providing androgen at its site of physiological production, metabolism and action with the avoidance of systemic exposure thus preventing or minimizing side-effects. Androgen concentrations in human follicular fluid, reflecting the physiological levels that granulosa cells and oocytes are exposed to, are much higher than in the circulation (Table 1). In antral follicles of 3–12 mm, the follicular fluid concentrations of testosterone and androstenedione are 200–350 nmol/L and 2,000–3,800 nmol/L, respectively (Table 2) (21). The concentration of testosterone in the circulation in pre-menopausal women is around 1 nmol/l, while it is 20 times higher in the circulation of men with symptoms of androgen deficiency occurring in men below ~10 nmol/l. For comparison, the intratesticular concentration of testosterone is around 1,000 nmol/l (25).

**Table 1:** Concentrations of androgens in circulation of women and men as compared to that of follicle fluid in human small antral follicles with a diameter of 3−9 millimeter (*N* = 545 for testosterone and *N* = 285 for androstenedione).

	**Testosterone**	**Androstenedione**
	**(nmol/L)**	**(nmol/L)**
Women pre-menopausal^*^	0.5–1.5	5–9
Men	10–35	3–7
Human follicular fluid (mean; median)	245 [194]	2,361 [2,256]

* *Data in circulation from (24)*.

**Table 2:** Distribution of testosterone and androstenedione concentrations in fluid from human small antral follicles with a diameter between 3 and 10 mm obtained in women between 17 and 43 years of age (*N* = 545 for testosterone and *N* = 285 for androstenedione).

	**Testosterone concentration (nmol/L)**							
Testosterone(nmol/l)	<50	50–100	100–200	200–300	300–400	400–500	500–600	>600
No. follicles	37	91	171	100	75	50	21	33
% follicles	7	17	31	18	14	9	4	6
	**Androstenedione concentration (nmol/L)**							
Androstenedione (nmol/l)	<100	100–500	500–1,000	1,000–1,500	1,500–2,000	2,000–3,000	3,000–4,000	>4,000
No. follicles	4	23	33	41	26	66	56	36
% follicles	1	8	12	14	9	23	20	13

Collectively, these data suggest that exogenous administration of testosterone is unlikely to affect follicular fluid concentrations of androgens to which granulosa cells are physiologically exposed without inducing virilizing side-effects. Additionally, high doses of testosterone will exert negative feedback effects on gonadotrophin secretion, which will certainly impact follicle development and function.

To support the notion that that administration of exogenous androgens acting through the systemic circulation would exert little if any effect on the local intrafollicular environment affecting granulosa cells, it was recently shown that there was no correlation between serum testosterone levels and follicular fluid follicular fluid testosterone, DHEA, estradiol or anti Müllerian Hormone (AMH) concentrations (26). This confirms that intrafollicular sex-steroid concentrations in individual follicles are unlikely to be affected via the circulation.

## Effect of Androgen Administration in Female to Male Transgender Patients

Female to male transgender patients receive masculinizing doses of testosterone, which result in both physical and psychological effects (27, 28). These patients are often treated with around 200 mg testosterone intramuscularly every 2nd week or testosterone gels delivering 5–10 mg daily (27). These doses are sufficient to stop menstrual cycles and in some studies the ovaries have been described as having a polycystic ovary morphology (PCOM) (29–31). When compared to ovaries from regularly cycling women, the ovaries of transmen were enlarged, with a 2-fold increase in the number of cystic antral follicles, 3.5-fold increase in atretic follicles, a collagenized 3-fold thicker cortex, hyperplasia of the theca interna and stroma, and clusters of luteinized stromal cells (31). However, in a recent paper, the prevalence of PCOM was not significantly increased after long-term treatment of transmen with testosterone (32). This undoubtedly results from direct effects on the ovaries but is complicated by endocrinological effects on the pituitary with reduced secretion of LH and FSH. This group of patients clearly demonstrate that androgen priming with administration of exogenous testosterone in low-responder women may have complex ovarian and systemic effects, and it may be difficult to determine a dose that provides additional recruitment of follicles without inducing unwanted side effects elsewhere in the body.

## Clinical Trials Investigating Androgen Priming by Use of Exogenous Androgens

Dehydroepiandrosterone sulfate (DHEAS), DHEA, androstenedione, testosterone and dihydrotestosterone (DHT) are the primary circulating androgens in women of reproductive age. Only testosterone and DHT are potent androgens, whereas the others are precursors that require conversion in order to exert stronger androgenic effects (33).

DHEA is a precursor of both testosterone and estradiol. It is produced in the adrenal Zona Reticularis (50%), ovarian stromal cells (20%), and from conversion of DHEAS (30%). DHEA is a weak partial agonist of the AR. It exerts androgenic effects *via* conversion into androstenedione and testosterone. It is also a prohormone in the synthesis of estrogens *via* conversion by aromatase of testosterone and androstenedione to estrone and estradiol (34).

Gleicher et al. (35) proposed that some forms of DOR could be caused by “adrenal hypoandrogenism.” Patients with DOR and adrenal hypoandrogenism are generally younger and have low-normal levels of DHEA. In contrast, they proposed that older women with DOR can have what they termed functional hypoandrogenism due to reduced conversion of DHEA to testosterone by the theca cells. Their serum DHEA levels are increased due to reduced conversion. They hypothesize that the patient most likely to respond to DHEA supplementation will be the former, younger patient with adrenal hypoandrogenism, whereas the latter might benefit more from supplementation with testosterone. This has not, however, been confirmed, and the potential diagnosis of “adrenal hypoandrogenism” is unclear (35).

### DHEA

DHEA is widely used as pretreatment for low responders undergoing IVF in order to increase the number of growing follicles. A Cochrane review in 2015 investigated the effect of DHEA supplementation on IVF outcome. This meta-analysis showed a small positive effect on clinical pregnancy rate [Odds ratio (OR) 1,34; CI 1.01–1.76, 12 randomized controlled trials (RCTs)] and live birth rate (OR 1.81; CI 1.25–2.62, 8 RCTs). A funnel plot gave no indication of publication bias (36). The most recent RCT of DHEA supplementation for IVF is from 2016. These studies are summarized in Table 3. However, the patients included are heterogeneous between studies; they varied from infertile women with normal ovarian reserve markers to older women with severe DOR. It is possible that DHEA supplementation might be beneficial in subgroups of women with DOR, but DHEA adjuvant treatment should be considered experimental. Currently, a further RCT investigating adjuvant DHEA (DITTO study) is awaited.

**Table 3:** Outcome of RCTs in which DHEA was used for supplementation in IVF treatment.

**Study**	**Design**	**No. of patients**	**DHEA regime**	**Conclusion clinical pregnancy**
Artini et al. (37)	DHEA vs. no co-treatment	24, poor responders, Bologna criteria, age 31–42	Daily oral DHEA 25 mg × 3 daily, starting 3 months prior to IVF	DHEA 3/12 vs. control 2/12
Divita et al. (38), conference abstract only	DHEA vs. placebo	31, infertile, poor responders, age?	Daily oral 40 mg micronized DHEAS, starting CD 21 in previous cycle, GnRH agonist protocol	DHEA 7/16 vs. control 5/15
Evans et al. (39), conference abstract only	DHEA vs. placebo	41 Poor responders	Daily DHEA oral 75 mg × 1, for 4 months prior to IVF	DHEA 1/21 vs. control 1/20 Live Birth: DHEA 0/21 vs. control 1/20
Jindal et al. (40), conference abstract only	DHEA vs. no co-treatment	406, poor responders	Daily micronized DHEA oral 75 mg for up to 6 months, combination of GnRH agonist and antagonist cycles	DHEA 39/203 vs. control 20/203 Live Birth: DHEA 35/203 vs. control 17/203
Kara et al. (41)	DHEA vs. no co-treatment	208, diminished ovarian reserve	Daily oral DHEA 25 mg × 3 daily, starting 3 months prior to IVF	DHEA 33/104 vs. control 34/104
Moawad et al. (42)	DHEA vs. no co-treatment	133, poor responders	Daily oral DHEA 25 mg × 3 daily, starting 3 months prior to IVF	DHEA 12/67 vs. control 8/66 Live Birth: DHEA 11/67 vs. control 7/66
Tartagni et al. (43)	DHEA vs. placebo	52 infertile but not poor responders	Daily DHEA oral 75 mg × 1, starting 8 weeks before IVF	DHEA 10/26 vs. control 7/26 Live Birth: DHEA 10/26 vs. control 4/26
Tartagni et al. (44)	DHEA vs. placebo	109 infertile, undergoing first IVF cycle	Daily DHEA oral 75 mg × 1, starting 8 weeks before IVF	DHEA 22/53 vs. control 18/56 Live Birth: DHEA 22/53 vs. control 13/56
Wiser et al. (45)	DHEA vs. no co-treatment	33, diminished ovarian reserve	Daily oral DHEA 75 mg × 1 for at least 6 weeks prior to IVF	DHEA 4/17 vs. control 2/16 Live Birth: DHEA 3/17 vs. control 1/16
Yeung et al. (46), conference abstract only	DHEA vs. placebo	72, normal responders	Daily DHEA oral 25 mg × 3, 12 weeks prior to IVF	DHEA 10/36 vs. control 15/36 Live Birth: DHEA 7/36 vs. control 11/36
Yeung et al. (47)	DHEA vs. placebo	32, expected poor response	Daily DHEA oral 25 mg × 3, 12 weeks prior to IVF	DHEA 3/16 vs. control 4/16 Live Birth: DHEA 2/16 vs. control 2/16
Zhang et al. (48)	DHEA vs. no co-treatment	105, dimished ovarian reserve	Daily DHEA oral 25 mg × 3, 12 weeks prior to IVF	DHEA 8/52 vs. control 7/53
Kotb et al. (49)	DHEA vs. no co-treatment	140, poor responders, Bologna criteria	Daily DHEA oral 25 mg × 3, 12 weeks prior to IVF	DHEA 20/70 vs. control 9/70
**Other non-RCTs on DHEA supplementation are listed in this table**				
**Study**	**Design**	**No. of patients**	**DHEA regime**	**Conclusion**
Barad et al. (50)	Case-Control	165, diminished ovarian reserve	Daily DHEA oral 25 mg × 3, up to 4 months prior to IVF	DHEA 13/64 vs. control 11/101
Xu et al. (51)	Retrospective cohort study	386, poor responders according to Bologna Criteria	Daily DHEA oral 25 mg × 3, 12 weeks prior to IVF	DHEA 57/189 vs. control 37/197
Vlahos et al. (52)	Prospective cohort study	161, poor responders	Daily DHEA oral 25 mg × 3, 12 weeks prior to IVF	DHEA 1/48 vs. control 8/113
Chern et al. (53)	Retrospective cohort study	151 poor responders, Bologna Criteria	90 mg oral DHEA daily.	DHEA 16/67 vs. control 6/84 Live Birth: DHEA 12/67 vs. control 5/84

### Testosterone

The aim of using testosterone as an adjuvant treatment in women with a low ovarian reserve undergoing OS is to increase the number of follicles which are sensitive to gonadotropin and thereby achieve more mature follicles, more collected oocytes, and thus increase the pregnancy and live birth rates.

Exogenous testosterone for androgen priming is usually applied either transdermally by administration of a testosterone gel (most frequently) or a patch, or through oral capsules. The doses and duration of treatment varies between studies. A recent systematic review and meta-analysis of 7 RCTs including women with a poor ovarian response undergoing IVF published from 2006 to 2018 found that treatment with testosterone increased the number of oocytes collected and most importantly the live birth rate (RR 2.29, 95% CI 1.31–4.01, *p* = 0.004), with low statistical heterogeneity between the included studies (54).

The testosterone doses used in the treatment arms of the 7 RCTs were 10–25 mg/day (gel) (55–58), 2.5 mg/day (patch) (59), or 40 mg/day (oral capsules) (60). The timing and the duration of the testosterone treatment varied from five to 51 days preceding OS, or only during the rFSH stimulation period in one pilot RCT (61).

Despite the use of androgens as an adjuvant for OS being off label, it is used by many physicians for treatment of women with a DOR (62), and the range of protocols identified (54) indicates that the evidence for the use of priming with testosterone before OS is not robust. The ongoing T-TRANSPORT trial investigates if 5.5 mg testosterone/day (gel) for 2 months as a pretreatment to OS will result in more pregnancies compared to placebo in women with DOR (63). The safety of these relatively high doses also needs to be established.

## Clinical Trials Investigating Androgen Priming by Use of hCG, LH, OR AIs

A concept of short-term local, intra-ovarian “androgen priming” *before* OS was developed and investigated in expected normal responder women with regular menstrual cycles (64, 65). HCG (LH activity) and an AI were administered to achieve a temporary enhancement of local androgen exposure (within the follicles) before OS. Early follicular phase down-regulation using a GnRH-antagonist was applied from cycle day (CD) two and during the priming period, before initiation of OS with gonadotropins, to inhibit development of a dominant follicle. The control group received only the GnRH antagonist during the priming period. The treatment regimen was successful in raising intrafollicular androgen levels in pre-ovulatory follicular fluids collected during oocyte retrieval almost 2 weeks after cessation of the priming period, with significantly higher testosterone levels compared to controls [26.4 nmol/l (95%CI 24.6–27.6) vs. 22.4 nmol/l (95%CI 19.3–22.4), *p* = 0.014] (65). Furthermore, the proportion of the antral follicle count on CD2 that reached the preovulatory state, and the serum estradiol level per preovulatory follicle, were higher in the priming group in the larger study, however, no real clinical benefit was observed (65).

Another group performed an RCT (*n* = 147) including pretreatment with rLH for seven days (300 IU per day) administered during GnRH-agonist downregulation in the long protocol just before initiation of rFSH stimulation (66). The rLH pretreatment significantly increased the number of small antral follicles, as compared to the untreated group. At stimulation day one, the plasma LH level was significantly higher in the rLH pretreatment group, but estradiol and testosterone levels were similar between groups, then and at stimulation day 8. Significantly more fertilized embryos were seen in the rLH pretreated group as well as a trend toward more high-grade embryos. The ongoing pregnancy rate was similar. Follicular fluid analyses were not performed.

In the above-mentioned studies expected normal responders with regular menstrual cycles rather than poor-responders were included, and androgen priming was used in the short-term (days) rather than long-time (weeks to months).

Bercaire and coworkers (67) investigated an “ANDRO-IVF protocol” including a 24-days pretreatment in poor responder patients (*n* = 13) who had undergone a failed IVF cycle, which was later used as the control. From CD one transdermal AndroGel(r) (67) 25 mg every other day, oral letrozole 2.5 mg daily, and hCG 2500 IU subcutaneously twice a week were administered. Cycle control was performed with estradiol valerate from CD three to CD 15 followed by micronized progesterone from CD 15 to CD 24, followed by a new menstrual cycle, in which high dose OS and IVF took place. They reported a significantly lower cancellation rate, a higher number of oocytes and metaphase II oocytes, a higher fertilization rate and a higher number of embryos per patient and concluded that the protocol seemed to improve clinical outcomes. However, the small sample size, the use of higher daily gonadotropin doses and longer duration of OS, as well-including failed cycles only as controls (with a known tendency of regression toward the mean) limit firm conclusions, and as the authors state, further RCTs are needed (67).

Supplementation with hCG activity or aromatase inhibitors *during* OS for IVF has also been explored. Different doses of hCG (0–150 IU daily) *during* rFSH stimulation were explored in an RCT using the long GnRH agonist protocol (68). Patients were stratified into three groups according to the concentration of serum hCG at steady state at Day 6 of stimulation (<3.5 IU/l, 3.5–8.0 IU/l and >8.0 IU/l), and the mean numbers of top-quality embryos were 0.5+0.9, 1.1+1.8, and 1.5+1.5, respectively (*P* = 0.03). Cumulative live birth rates including subsequent frozen embryo replacement (FER) cycles were similar, but the study was not powered to detect potential differences in pregnancy or delivery rates.

Following a long GnRH agonist protocol, OS with Highly Purified human Menopausal Gonadotropin (HP-hMG) containing hCG activity (~30 IU/day) was compared to rFSH in an RCT (69). Among the HP-hMG-treated patients, those with the highest day 6 hCG levels had the highest ongoing pregnancy rates. Also, the number of top-quality embryos and the proportion of patients with top-quality embryos in the HP-hMG group were positively correlated with circulating levels of hCG. The fertilization rate was similar in the HP-hMG and rFSH group, but the proportion of top-quality embryos was higher with HP-hMG. The serum level of hCG and androgens were higher at day 6 and at the end of stimulation in the HP-hMG group as were the levels in pre-ovulatory follicular fluid.

The use of an AI during OS for IVF treatment was included in a recent Cochrane review (70). There was no conclusive evidence indicating that treatment with letrozole with or without gonadotropins differed from gonadotropins alone in GnRH-agonist or antagonist protocols with respect to effects on live-birth or pregnancy rates, either in the general population of women undergoing IVF treatment or in poor responders. Larger, high-quality RCTs are needed to reach a firm conclusion as to whether this approach should be adopted into routine clinical practice.

## Long-Term Low Dose hCG Priming: Local Increased Androgen Levels

It can be argued that, albeit simplifying the complex pathophysiology of the condition, women with PCOS experience long-term androgen priming with increased endogenous concentrations of LH that result in multiple small antral follicles, which may be recruited in response to OS with gonadotropins and result in the well-known excessive response that characterizes these patients. Potentially, a long-term low dose hCG administration could result in a phenotype resembling PCOS at least in terms of an increased recruitment (or accumulation) of small antral follicles, which could lead to an increased harvest of mature oocytes in response to OS. Thus, the concept of creating a reversible PCO-like condition could be of interest in connection with poor responder women.

A first attempt to achieve this has recently been published (71) in which low responder women (*n* = 30) received 260 IU hCG every 2nd day for 8 weeks to augment intra-ovarian androgen levels. Simultaneously women received 2.5 mg letrozole daily to prevent androgens from being converted into estrogens, and in addition women were down regulated with a GnRH agonist to prevent FSH from rising in response to low levels of estradiol. Priming stopped on the 1st day of OS. The primary endpoint was circulating concentration of AMH after 8 weeks of priming, while secondary endpoints included antral follicle count (2–10 mm), and serum hCG, testosterone and progesterone levels (71). However, the intervention did not produce any significant improvements, and the resulting androgen production was lowered compared to the natural situation. This is likely to be because the administration of hCG was unable to overcome the lowering of gonadotropin levels caused by GnRH agonist administration. Further studies addressing this issue need to explore other androgen priming protocols and their clinical value in IVF.

## Conclusions

In the human ovary, AR is expressed from the primary and primordial stage onwards with all multi-layered preantral follicles expressing AR, thus androgens are likely to have important physiological roles at all stages of folliculogenesis. The positive correlation between follicular fluid androgen levels and granulosa cell FSHR expression indicates that androgens promote follicular responsiveness to FSH. There is some experimental evidence to support this, in addition to the recognized hypersensitivity to exogenous FSH of women with PCOS, but the difficulties of studying human folliculogenesis mean that many questions regarding the roles of androgens in the ovary remain unanswered. It is important to recognize that androgen concentrations in follicular fluid by far exceed the levels in the female circulation, thus while exogenous administration of androgens may have effects on early stages of follicle development, it is unlikely to affect concentrations of androgens in more mature follicles. Conversely, administration of hCG or an AI does increase intra-follicular androgen concentration and may have effects across a broader range of stage of follicle development. Current clinical evidence regarding administration of exogenous DHEA or testosterone seems to increase live birth rates, but more robust studies are required to confirm this, and optimize protocols: at present, such adjuvant treatment should therefore be considered experimental. Currently, two RCTs investigating adjuvant DHEA (DITTO study) and transdermal testosterone (T-TRANSPORT) are awaited and their results will shed light on this potential therapeutic approach.

## Author Contributions

CA, KL, and RA conceived the idea. KL, NF, MW, RA, and CA collected data. KL, NF, MW, KB, MH, LM, RA, and CA wrote and approved the paper. All authors contributed to the article and approved the submitted version.

## Conflict of Interest

The authors declare that the research was conducted in the absence of any commercial or financial relationships that could be construed as a potential conflict of interest.

## References

[1] Hillier SG Tetsuka M Fraser HM. Local and developmental regulation of androgen receptor in primate ovary. Hum Reprod. (1997) 12:107–11. 9043913 10.1093/humrep/12.1.107

[2] Franks S Hardy K. Androgen action in the ovary. Front Endocrinol. (2018) 9:452. 10.3389/fendo.2018.00452 30147675 PMC6097027

[3] Dilaver N Pellatt L Jameson E Ogunjimi M Bano G Homburg R. The regulation and signalling of anti-Müllerian hormone in human granulosa cells: relevance to polycystic ovary syndrome. Hum Reprod. (2019) 34:2467–79. 10.1093/humrep/dez214 31735954

[4] Owens LA Kristensen SG Lerner A Christopoulos G Lavery S Hanyaloglu AC Gene expression in granulosa cells from small antral follicles from women with or without polycystic ovaries. J Clin Endocrinol Metab. (2019) 104:6182–92. 10.1210/jc.2019-00780 31276164 PMC6822643

[5] Agarwal SK Judd HL Magoffin DA. A mechanism for the suppression of estrogen production in polycystic ovary syndrome. J Clin Endocrinol Metab. (1996) 81:3686–91. 10.1210/jcem.81.10.8855823 8855823

[6] Yang F Ruan YC Yang YJ Wang K Liang SS Han YB. Follicular hyperandrogenism downregulates aromatase in luteinized granulosa cells in polycystic ovary syndrome women. Reproduction. (2015) 150:289–96. 10.1530/REP-15-0044 26199450

[7] Da Broi MG Giorgi VSI Wang F Keefe DL Albertini D Navarro PA. Influence of follicular fluid and cumulus cells on oocyte quality: clinical implications. J Assist Reprod Genet. (2018) 35:735–51. 10.1007/s10815-018-1143-3 29497954 PMC5984887

[8] Nielsen ME Rasmussen IA Kristensen SG Christensen ST Møllgård K Wreford Andersen E. In human granulosa cells from small antral follicles, androgen receptor mRNA and androgen levels in follicular fluid correlate with FSH receptor mRNA. Mol Hum Reprod. (2011) 17:63–70. 10.1093/molehr/gaq073 20843821

[9] Rice S Ojha K Whitehead S Mason H. Stage-specific expression of androgen receptor, follicle-stimulating hormone receptor, and anti-Müllerian hormone type II receptor in single, isolated, human preantral follicles: relevance to polycystic ovaries. J Clin Endocrinol Metab. (2007) 92:1034–40. 10.1210/jc.2006-1697 17179193

[10] Jeppesen JV Kristensen SG Nielsen ME Humaidan P Dal Canto M Fadini R. LH-receptor gene expression in human granulosa and cumulus cells from antral and preovulatory follicles. J Clin Endocrinol Metab. (2012) 97:E1524–31. 10.1210/jc.2012-1427 22659248 PMC3410279

[11] Saunders PTK Millar MR Macpherson S Harkiss D Anderson RA Orr B. Differential expression of estrogen receptor-a and -b and androgen receptor in the ovaries of marmoset and human. Biol Reprod. (2000) 63:1098–105. 10.1095/biolreprod63.4.1098 10993832

[12] Gougeon A. Regulation of ovarian follicular development in primates: facts and hypotheses. Endocr Rev. (1996) 17:121–55. 10.1210/edrv-17-2-121 8706629

[13] Leijdekkers JA Eijkemans MJC van Tilborg TC Oudshoorn SC van Golde RJT Hoek A. Cumulative live birth rates in low-prognosis women. Hum Reprod. (2019) 34:1030–41. 10.1093/humrep/dez051 31125412 PMC6555622

[14] Sen A Hammes SR. Granulosa cell-specific androgen receptors are critical regulators of ovarian development and function. Mol Endocrinol. (2010) 24:1393–403. 10.1210/me.2010-0006 20501640 PMC2903904

[15] Walters KA Simanainen U Handelsman DJ. Molecular insights into androgen actions in male and female reproductive function from androgen receptor knockout models. Hum Reprod Update. (2010) 16:543–58. 10.1093/humupd/dmq003 20231167

[16] Weil S Vendola K Zhou J Bondy CA. Androgen and follicle-stimulating hormone interactions in primate ovarian follicle development. J Clin Endocrinol Metab. (1999) 84:2951–6. 10.1210/jcem.84.8.5929 10443703

[17] Weil SJ Vendola K Zhou J Adesanya OO Wang J Bondy CA. Androgen receptor gene expression in the primate ovary: cellular localization, regulation, and functional correlations. J Clin Endocrinol Metab. (1998) 83:2479–85. 10.1210/jcem.83.7.4917 9661631

[18] Vendola KA Adesanya OO Weil SJ Bondy CA. Androgens stimulate early stages of follicular growth in the primate ovary. J Clin Invest. (1998) 101:2622–9. 10.1172/JCI2081 9637695 PMC508852

[19] Vendola KA Zhou J Wang J Famuiya OA Bierre M Bondy CA. Androgens stimulate primordial follicle development in the primate ovary. Biol Reprod. (1999) 61:353–7. 10.1095/biolreprod61.2.353 10411511

[20] Hillier SG Yong EL Illingworth PJ Baird DT Schwall RH Mason AJ. Effect of recombinant inhibin on androgen synthesis in cultured human thecal cells. Mol Cell Endocrinol. (1991) 75:R1–6. 10.1016/0303-7207(91)90234-J 2050269

[21] Kristensen SG Mamsen LS Jeppesen JV Bøtkjær JA Pors SE Borgbo T. Hallmarks of human small antral follicle development: implications for regulation of ovarian steroidogenesis and selection of the dominant follicle. Front Endocrinol. (2018) 8:376. 10.3389/fendo.2017.00376 29375481 PMC5770355

[22] Poulsen LC Englund ALM Andersen AS Bøtkjær JA Mamsen LS Damdimopoulou P. Follicular hormone dynamics during the midcycle surge of gonadotropins in women undergoing fertility treatment. Mol Hum Reprod. (2020) 26:256–68. 10.1093/molehr/gaaa013 32023345

[23] Yding Andersen C. Inhibin-B secretion and FSH isoform distribution may play an integral part of follicular selection in the natural menstrual cycle. Mol Hum Reprod. (2017) 23:16–24. 10.1093/molehr/gaw070 27756855

[24] Braunstein GD Reitz RE Buch A Schnell D Caulfield MP. Testosterone reference ranges in normally cycling healthy premenopausal women. J Sex Med. (2011) 8:2924–34. 10.1111/j.1743-6109.2011.02380.x 21771278

[25] Coviello AD Bremner WJ Matsumoto AM Herbst KL Amory JK Anawalt BD Intratesticular testosterone concentrations comparable with serum levels are not sufficient to maintain normal sperm production in men receiving a hormonal contraceptive regimen. J Androl. (2004) 25:931–8. 10.1002/j.1939-4640.2004.tb03164.x 15477366

[26] von Wolff M Stute P Eisenhut M Marti U Bitterlich N Bersinger NA Serum and follicular fluid testosterone concentrations do not correlate, questioning the impact of androgen supplementation on the follicular endocrine milieu. Reprod Biomed Online. (2017) 35:616–23. 10.1016/j.rbmo.2017.07.012 28821386

[27] Irwig MS Testosterone therapy for transgender men. Lancet Diabetes Endocrinol. (2017) 5:301–11. 10.1016/S2213-8587(16)00036-X 27084565

[28] Costa LBF Rosa-E-Silva ACJS Medeiros SF Nacul AP Carvalho BR Benetti-Pinto CL. Recommendations for the use of testosterone in male transgender. Rev Bras Ginecol Obstet. (2018) 40:275–80. 10.1055/s-0038-1657788 29913543 PMC10316880

[29] Futterweit W Deligdisch L. Histopathological effects of exogenously administered testosterone in 19 female to male transsexuals. J Clin Endocrinol Metab. (1986) 62:16–21. 10.1210/jcem-62-1-16 3940265

[30] Spinder T Spijkstra JJ van den Tweel JG Burger CW van Kessel H Hompes PG. The effect of long term testosterone administration on pulsatile luteinizing hormone secretion and on ovarian histology in eugonadal female to male transsexual subjects. J Clin Endocrinol Metab. (1989) 69:151–7. 10.1210/jcem-69-1-151 2471710

[31] Pache TD Chadha S Gooren LJG Hop WCJ Jaarsma KW Dommerholt HBR. Ovarian morphology in long-term androgen-treated female to male transsexuals. A model for the study of polycystic ovarian syndrome? Histopathology. (1991) 19:445–52. 10.1111/j.1365-2559.1991.tb00235.x 1757084

[32] Caanen MR Schouten NE Kuijper EAM van Rijswijk J van den Berg MH van Dulmen-den Broeder E. Effects of long-term exogenous testosterone administration on ovarian morphology, determined by transvaginal (3D) ultrasound in female-to-male transsexuals. Hum Reprod. (2017) 32:1457–64. 10.1093/humrep/dex098 28505246

[33] Burger HG. Androgen production in women. Fertil Steril. (2002) 77(Suppl.4):3–5. 10.1016/S0015-0282(02)02985-0 12007895

[34] Davison SL Davis SR. Androgens in women. J Steroid Biochem Mol Biol. (2003) 85:363–66. 10.1016/S0960-0760(03)00204-8 12943723

[35] Gleicher N Kim A Weghofer A Kushnir VA Shohat-Tal A Lazzaroni E. Hypoandrogenism in association with diminished functional ovarian reserve. Hum Reprod. (2013) 28:1084–91. 10.1093/humrep/det033 23427231

[36] Nagels HE Rishworth JR Siristatidis CS Kroon B. Andogens (dehydroepiandrosterone or testosterone) for women undergoing assisted reproduction. Cochrane Syst Rev. (2015) 11:CD009749. 10.1002/14651858.CD009749.pub2 26608695 PMC10559340

[37] Artini PG Simi G Ruggiero M Pinelli S Di Berardino OM Papini F. DHEA supplementation improves follicular microenviroment in poor responder patients. Gynecol Endocrinol. (2012) 28:669–73. 10.3109/09513590.2012.705386 22835219

[38] Divita AE Kanzepolsky LS Notrica JA Neuspiller FD Polak de Fried E Does dehidroepiandrosterone sulfate (Dheas) co-treatment improve art outcome? A prospective, randomized, double-blind placebo-controlled trial. Fertil Steril. (2003) 80(Suppl.3):S111–2. 10.1016/S0015-0282(03)02094-6

[39] Evans J A randomised, double blinded, placebo controlled, single centre trial comparing dehydroepiandrosterone (DHEA) and placebo in the treatment of women with resistant ovaries prior to *in vitro* fertilisation (IVF) and intra cytoplasmic sperm injection (ICSI). RCOG World Congress; 2013 June 24-26; Liverpool, UK. BJOG. (2013) EP2.93:225.

[40] Jindal A Singh R A prospective randomised controlled study on the role of dehydroepiandrosterone (DHEA) on improving ovarian response in known poor responders in previous failed IVF-ICSI cycles. Hum Reprod. (2014) 29(Supp.1):i14 10.3109/09513590.2013.824957

[41] Kara M Aydin T Aran T Turktekin N Ozdemir B. Does dehydroepiandrosterone supplementation really affect IVF-ICSI outcome in women with poor ovarian reserve? Eur J Obstetr Gynecol Reprod Biol. (2014) 173:63–5. 10.1016/j.ejogrb.2013.11.008 24331115

[42] Moawad M Shaeer M Long-term androgen priming by use of dehydroepiandrosterone (DHEA) improves IVF outcome in poor-responder patients. A randomized controlled study. Middle East Fertil Soc J. (2012) 17:268–74. 10.1016/j.mefs.2012.11.002

[43] Tartagni M Pergola G Damiani GR Pellegrino A Baldini D Tartagni MV. Potential benefit of dehydroepiandrosterone supplementation for infertile but not poor responder patients in a IVF program. Minerva Ginecol. (2015) 67:7–12. 24867068

[44] Tartagni M Cicinelli MV Baldini D Tartagni MV Alrasheed H DeSalvia MA. Dehydroepiandrosterone decreases the age-related decline of the *in vitro* fertilization outcome in women younger than 40 years old. Reprod Biol Endocrinol. (2015) 13:13–8. 10.1186/s12958-015-0014-3 25884390 PMC4355976

[45] Wiser A Gonen O Ghetler Y Shavit T Berkovitz A Shulman A. Addition of dehydroepiandrosterone (DHEA) for poor-responder patients before and during IVF treatment improves the pregnancy rate: a randomized prospective study. Hum Reprod. (2010) 25:2496–500. 10.1093/humrep/deq220 20729538

[46] Yeung TWY Li RHW Lee VCY Chai J Ng EHY Ho PC. A randomized double-blinded placebo-controlled trial on the effect of 16 weeks of dehydroepiandrosterone (DHEA) on ovarian reserve markers and IVF outcomes in normal responders. Fertil Steril. (2013) 100(3Suppl.):S114. 10.1016/j.fertnstert.2013.07.165823144466

[47] Yeung TW Chai J Li RH Lee VC Ho PC Ng EH. A randomized, controlled, pilot trial on the effect of dehydroepiandrosterone on ovarian response markers, ovarian response, and *in vitro* fertilization outcomes in poor responders. Fertil Steril. (2014) 102:108–15.e1. 10.1016/j.fertnstert.2014.03.044 24796766

[48] Zhang HH Xu PY Wu J Zou WW Xu XM Cao XY. Dehydroepiandrosterone improves follicular fluid bone morphogenetic protein-15 and accumulated embryo score of infertility patients with diminished ovarian reserve undergoing *in vitro* fertilization: a randomized controlled trial. J Ovarian Res. (2014) 7:93. 10.1186/s13048-014-0093-3 25330837 PMC4210503

[49] Kotb MM Hassan AM AwadAllah AM. Does dehydroepiandrosterone improve pregnancy rate in women undergoing IVF/ICSI with expected poor ovarian response according to the Bologna criteria? A randomized controlled trial. Eur J Obstet Gynecol Reprod Biol. (2016) 200:11–15. 10.1016/j.ejogrb.2016.02.009 26963897

[50] Barad D Brill H Gleicher N. Update on the use of dehydroepiandrosterone supplementation among women with diminished ovarian function. J Assist Reprod Genet. (2007) 24:629–34. 10.1007/s10815-007-9178-x 18071895 PMC3454995

[51] Xu B Li Z Yue J Jin L Li Y Ai J. Effect of dehydroepiandrosterone administration in patients with poor ovarian response according to the Bologna criteria. PLoS ONE. (2014) 9:e99858. 10.1371/journal.pone.0099858 24932478 PMC4059703

[52] Vlahos N Papalouka M Triantafyllidou O Vlachos A Vakas P Grimbizis G. Dehydroepiandrosterone administration before IVF in poor responders: a prospective cohort study. Reprod Biomed Online. (2015) 30:191–6. 10.1016/j.rbmo.2014.10.005 25498594

[53] Chern CU Tsui KH Vitale SG Chen S-N Wang P-H Cianci A. Dehydroepiandrosterone (DHEA) supplementation improves *in vitro* fertilization outcomes of poor ovarian responders, especially in women with low serum concentration of DHEA-S: a retrospective cohort study. Reprod Biol Endocrinol. (2018) 16:90. 10.1186/s12958-018-0409-z 30223902 PMC6142344

[54] Noventa M Vitagliano A Andrisani A Blaganje M Viganò P Papaelo E. Testosterone therapy for women with poor ovarian response undergoing IVF: a meta-analysis of randomized controlled trials. J Assist Reprod Genet. (2019) 36:673–83. 10.1007/s10815-018-1383-2 30610664 PMC6505000

[55] Massin N Cedrin-Durnerin I Coussieu C Galey-Fontaine J Wolf JP Hugues J-N. Effects of transdermal testosterone application on the ovarian response to FSH in poor responders undergoing assisted reproduction technique?a prospective, randomized, double-blind study. Hum Reprod. (2006) 21:1204–11. 10.1093/humrep/dei481 16476678

[56] Kim CH Howles CM Lee HA. The effect of transdermal testosterone gel pretreatment on controlled ovarian stimulation and IVF outcome in low responders. Fertil Steril. (2011) 95:679–83. 10.1016/j.fertnstert.2010.07.1077 20801436

[57] Kim CH Ahn JW Moon JW Kim SH Chae HD Kang BM. Ovarian features after 2 weeks, 3 weeks and 4 weeks transdermal testosterone gel treatment and their associated effect on IVF outcomes in poor responders. Dev Reprod. (2014) 18:145–52. 10.12717/DR.2014.18.3.145 25949183 PMC4282212

[58] Bosdou JK Venetis CA Dafopoulos K Zepiridis L Chatzimeletiou K Anifandis G. Transdermal testosterone pretreatment in poor responders undergoing ICSI: a randomized clinical trial. Hum Reprod. (2016) 31:977–85. 10.1093/humrep/dew028 26956551

[59] Fábregues F Penarrubia J Creus M Manau D Casals G Carmona F. Transdermal testosterone may improve ovarian response to gonadotrophins in low-responder IVF patients: a randomized, clinical trial. Hum Reprod. (2009) 24:349–59. 10.1093/humrep/den428 19054777

[60] Mskhalaya G Eltsova E Malysheva M Lubimkina E Zaletova V Kalinchenko S Testosterone undecanoate treatment in women with poor ovarian response undergoing IVF: pregnancy and live birth rates. Fertil Steril. (2016) 106(3Suppl.):e198–9. 10.1016/j.fertnstert.2016.07.576

[61] Saharkhiz N Zademodares S Salehpour S Hosseini S Nazari L Tehrani HG. The effect of testosterone gel on fertility outcomes in women with a poor response in *in vitro* fertilization cycles: a pilot randomized clinical trial. J Res Med Sci. (2018) 23:3. 10.4103/jrms.JRMS_864_17 29456560 PMC5813298

[62] Andersen MF Drakopoulos P Humaidan P Gomez JL Bruna I Rombauts L Off-label use of androgens and letrozole in infertile women - a multinational survey in Europe and Australia. Hum Reprod. (2018) 33:499–500

[63] Montoya-Botero P Rodriguez-Purata J Polyzos NP. Androgen supplementation in assisted reproduction: where are we in 2019? Curr Opin Obstet Gynecol. (2019) 31:188–94. 10.1097/GCO.0000000000000532 30855291

[64] Lossl K Andersen AN Loft A Freiesleben NL Bangsbøll S Andersen CY. Androgen priming using aromatase inhibitor and hCG during early-follicular-phase GnRH antagonist down-regulation in modified antagonist protocols. Hum Reprod. (2006) 21:2593–600. 10.1093/humrep/del221 16785262

[65] Lossl K Andersen CY Loft A Freiesleben NL Bangsbøll S Andersen AN. Short-term androgen priming by use of aromatase inhibitor and hCG before controlled ovarian stimulation for IVF. A randomized controlled trial. Hum Reprod. (2008) 23:1820–9. 10.1093/humrep/den131 18487212

[66] Durnerin CI Erb K Fleming R Hillier H Hillier SG Howles CM. Effects of recombinant LH treatment on folliculogenesis and responsiveness to FSH stimulation. Hum Reprod. (2008) 23:421–6. 10.1093/humrep/dem388 18084048

[67] Bercaire L Nogueira SM Lima PC Alves VR Donadio N Dzik A. ANDRO-IVF: a novel protocol for poor responders to IVF controlled ovarian stimulation. JBRA Assist Reprod. (2018) 22:52–5. 10.5935/1518-0557.20180011 29303236 PMC5844660

[68] Thuesen LL Loft A Egeberg AN Smitz J Petersen JH Andersen AN. A randomized controlled dose-response pilot study of addition of hCG to recombinant FSH during controlled ovarian stimulation for *in vitro* fertilization. Hum Reprod. (2012) 27:3074–84. 10.1093/humrep/des256 22791754

[69] Smitz J Andersen AN Devroey P Arce JC MERIT Group Endocrine profile in serum and follicular fluid differs after ovarian stimulation with HP-hMG or recombinant FSH in IVF patients. Hum Reprod. (2007) 22:676–87. 10.1093/humrep/del445 17110397

[70] Kamath MS Maheshwari A Bhattacharya S Lor KY Gibreel A Oral medications including clomiphene citrate or aromatase inhibitors with gonadotropins for controlled ovarian stimulation in women undergoing *in vitro* fertilisation. Cochrane Database Syst Rev. (2017) 11:CD008528 10.1002/14651858.CD008528.pub3 29096046 PMC6486039

[71] Vuong LN Ho TM Ha AN Pham TD Le TTN Yding Andersen C. The effect of intra-ovarian androgen priming on ovarian reserve parameters in Bologna poor responders. Reprod Biomed Online. (2020) 40:223–8. 10.1016/j.rbmo.2019.11.005 31974029

